# Long-term SGLT2 inhibitor therapy improves myocardial strain and diastolic function in HFpEF: a retrospective study linking functional recovery to reduced myocardial fibrosis

**DOI:** 10.3389/fcvm.2026.1858364

**Published:** 2026-07-21

**Authors:** Xiaojin Xu, Hongyang Liu, Runyang Chen

**Affiliations:** Department of Cardiology, The Affiliated Huai′an NO. 1 People′s Hospital of Nanjing Medical University, Huai’an, Jiangsu, China

**Keywords:** diastolic function, heart failure with preserved ejection fraction, myocardial fibrosis, myocardial strain, sodium-glucose co-transporter 2 inhibitors

## Abstract

**Background:**

The pathophysiological mechanisms of heart failure with preserved ejection fraction (HFpEF) are complex, with myocardial fibrosis being one of its core features, closely associated with diastolic dysfunction and poor prognosis. Sodium-glucose co-transporter 2 inhibitors (SGLT2i) have shown clinical benefits in patients with HFpEF; however, their effects on myocardial strain and diastolic function, as well as their relationship with the reduction of myocardial fibrosis, remain unclear.

**Objective:**

To evaluate the effects of long-term SGLT2i treatment (≥6 months) on left ventricular global longitudinal strain (GLS), diastolic function parameters, and serum fibrosis markers in patients with HFpEF, and to explore the potential mechanisms, through a retrospective cohort study.

**Methods:**

A total of 185 patients with HFpEF diagnosed at our hospital between June 2023 and January 2025 were consecutively enrolled and divided into an SGLT2i group (*n* = 93) and a control group (*n* = 92) based on whether they received SGLT2i treatment. Demographic data, echocardiographic parameters (GLS, LVEF, E/e’, LAVI, LVMI, TRV), and serological indicators (NT-proBNP, PⅢNP, GDF-15) were collected at baseline and during follow-up (median 12 months). The primary endpoint was the change in GLS (*Δ*GLS); secondary endpoints included changes in diastolic function parameters, rates of biomarker change, and heart failure rehospitalization rate. Independent sample t-tests, Mann–Whitney U tests, and chi-square tests were used to compare intergroup differences; Pearson correlation analysis was performed to assess the correlation between *Δ*GLS and *Δ*PⅢNP, *Δ*NT-proBNP; multivariate linear regression analysis was used to identify independent influencing factors of GLS improvement; Kaplan–Meier method was used to evaluate rehospitalization-free survival.

**Results:**

Baseline characteristics were balanced and comparable between the two groups (all *P* > 0.05). After 6 months of treatment, GLS improved significantly (*Δ*GLS: −2.49% ± 0.54% vs. −0.44% ± 0.52%, *P* < 0.001). NT-proBNP decreased by 30.4% vs. 9.7% (*P* < 0.001), PⅢNP by 15.1% vs. 5.8% (*P* < 0.001), and GDF-15 by 20.5% vs. 8.6% (*P* < 0.001). During the follow-up period, the heart failure rehospitalization rate in the SGLT2i group was significantly lower than that in the control group (24.7% vs. 55.4%, *P* < 0.001), and rehospitalization-free survival was significantly higher (Log-rank *P* < 0.001). In the overall population, *Δ*GLS was significantly positively correlated with *Δ*PⅢNP (r = 0.554, *P* < 0.001). Multivariate regression analysis showed that SGLT2i treatment was an independent predictor of GLS improvement (*β*= −2.05, *P* < 0.001).

**Conclusion:**

Long-term SGLT2i treatment can significantly improve myocardial strain, diastolic function, and clinical outcomes in patients with HFpEF, and its effects are closely related to the reduction of myocardial fibrosis markers, suggesting that the anti-fibrotic effect may be an important mechanism of SGLT2i cardioprotection.

## 1 Introduction

Heart failure with preserved ejection fraction (HFpEF) has emerged as one of the most challenging clinical syndromes in cardiovascular medicine in the 21st century. Epidemiological data demonstrate that HFpEF accounts for approximately 50% of all heart failure cases, and its prevalence continues to rise steadily alongside the aging population, as well as the increasing prevalence of obesity and diabetes (1–3). In contrast to the breakthrough therapeutic advances achieved in heart failure with reduced ejection fraction (HFrEF) over the past few decades, HFpEF is characterized by more complex pathophysiological mechanisms, relatively limited effective therapeutic options, and no significant improvement in patient prognosis (4).

The pathophysiological mechanisms of HFpEF are highly heterogeneous, involving systemic inflammation, endothelial dysfunction, metabolic abnormalities, and multi-organ crosstalk (5). Consensus from recent studies indicates that HFpEF is essentially a multisystem disorder, in which the development of its cardiac phenotype is jointly influenced by dysfunction of multiple organs including the kidneys, lungs, liver, adipose tissue, and skeletal muscle (6–8). Within this complex network, myocardial fibrosis has been identified as one of the core features of cardiac remodeling in HFpEF (9, 10). Excessive deposition of extracellular matrix leads to increased ventricular stiffness, impaired diastolic function, and ultimately promotes the progression of heart failure. Histological studies have shown that more than 90% of HFpEF patients exhibit varying degrees of myocardial fibrosis, with approximately one-third presenting moderate to severe fibrotic changes (11). Left ventricular hypertrophy and myocardial fibrosis interact causally, collectively forming the structural basis of diastolic dysfunction in HFpEF patients, and are closely associated with reduced exercise tolerance and poor prognosis (12).

Quantitative detection of serum biomarkers provides a non-invasive approach for assessing myocardial fibrosis. The N-terminal propeptide of type III procollagen (PⅢNP), a serological marker of myocardial fibrosis, is elevated in response to active extracellular matrix collagen metabolism and is closely linked to adverse outcomes in HFpEF patients. Growth differentiation factor-15 (GDF-15) has also been proven to participate in myocardial fibrosis and inflammatory responses, serving as an independent predictor of prognosis in HFpEF (13). Dynamic changes in these biomarkers can indirectly reflect the progression of myocardial fibrosis and its response to therapeutic interventions, offering important insights into the therapeutic mechanisms of HFpEF.

Sodium-glucose cotransporter 2 inhibitors (SGLT2i) were initially developed as anti-hyperglycemic agents; however, large-scale clinical trials have confirmed their substantial benefits in patients with heart failure, including those with HFpEF. The EMPEROR-Preserved and DELIVER trials demonstrated that empagliflozin and dapagliflozin significantly reduced the composite endpoint of cardiovascular death or heart failure hospitalization in HFpEF patients (14). Nevertheless, the precise mechanisms underlying the prognostic improvement conferred by SGLT2i in HFpEF remain incompletely elucidated. Furthermore, their benefits in HFpEF are mainly manifested in reducing the risk of heart failure hospitalization rather than improving mortality (15, 16), suggesting that further investigation into their cardioprotective mechanisms is warranted.

Animal and cellular experiments have provided critical insights into the anti-fibrotic effects of SGLT2i. Studies have shown that SGLT2i attenuate myocardial fibrosis through multiple pathways: activating the AMPK signaling pathway, alleviating oxidative stress, and inhibiting pro-fibrotic signaling pathways such as TGF-*β*/Smad (17, 18); inhibiting the activity of sodium-hydrogen exchanger-1 (NHE-1) in cardiac fibroblasts and reducing the expression of transforming growth factor-*β*2 (19); improving mitochondrial function and autophagic balance, and mitigating inflammatory responses (20). These effects are also observed in non-diabetic models, indicating that their anti-fibrotic action is independent of glucose-lowering effects (21, 22). Notably, SGLT2 is expressed in both cardiomyocytes and cardiac endothelial cells, and its expression is upregulated under heart failure conditions (23–25), providing a molecular basis for the direct cardiac effects of SGLT2i.

To date, two dedicated systematic reviews and meta-analyses have specifically evaluated the anti-fibrotic effects of SGLT2 inhibitors in heart failure. A meta-analysis by Wang et al. (18) demonstrated that empagliflozin significantly reduces diffuse myocardial fibrosis as assessed by cardiac magnetic resonance extracellular volume mapping, with a pooled effect size of −1.48 (95% CI −1.76 to −1.21, *P* < 0.00001). Another systematic review and meta-analysis further confirmed that SGLT2 inhibitor therapy attenuates ventricular remodeling and reduces serum collagen biomarkers in patients with heart failure (26). Together, these meta-analyses provide robust quantitative evidence supporting the anti-fibrotic cardioprotection of SGLT2 inhibitors.

Clinical imaging studies further support the anti-fibrotic potential of SGLT2i. A recent prospective study using cardiac magnetic resonance imaging showed that in HFpEF patients with diabetes, treatment with SGLT2i for 6 months resulted in a significant reduction in myocardial extracellular volume (ECV), accompanied by improved left ventricular global longitudinal strain (GLS) (27). As an imaging indicator of diffuse myocardial fibrosis, the decrease in ECV suggests that SGLT2i may improve myocardial function by attenuating myocardial fibrosis (28). However, systematic studies on the effects of long-term SGLT2i therapy on myocardial fibrosis biomarkers, myocardial strain, and diastolic function parameters in HFpEF patients remain limited, particularly evidence in Asian populations.

Left ventricular global longitudinal strain (GLS) is a more sensitive indicator of myocardial systolic function than left ventricular ejection fraction (LVEF), enabling early detection of subclinical myocardial dysfunction and showing a strong correlation with prognosis in HFpEF patients. Diastolic function parameters such as the E/e’ ratio and left atrial volume index (LAVI) directly reflect the core pathophysiological alterations in HFpEF. Comprehensive evaluation of changes in these parameters, combined with quantitative detection of fibrosis biomarkers, will facilitate a deeper understanding of the cardioprotective mechanisms of SGLT2i in HFpEF.

Against this background, the present study employed a retrospective cohort design to investigate the effects of long-term SGLT2i therapy (≥6 months) on left ventricular myocardial strain and diastolic function parameters in HFpEF patients. Additionally, by quantitatively measuring serum fibrosis biomarkers including PⅢNP and GDF-15, we analyzed the correlation between improvements in myocardial function and attenuation of fibrosis. We hypothesized that SGLT2i could significantly enhance myocardial strain and diastolic function in HFpEF patients, and that this effect is closely associated with reduced myocardial fibrosis, thereby providing novel clinical evidence for the cardioprotective role of SGLT2i in the treatment of HFpEF.

## 2 Materials and methods

### 2.1 Study design and participants

This was a single-center, retrospective, case-control study. We consecutively enrolled adult patients (age ≥ 18 years) diagnosed with heart failure with preserved ejection fraction (HFpEF) and receiving regular follow-up at the Department of Cardiology of The Affiliated Huai’an NO. 1 People's Hospital of Nanjing Medical University between June 2023 and January 2025. The requirement for informed consent was waived by the ethics committee of The Affiliated Huai‘an No. 1 People's Hospital of Nanjing Medical University due to the retrospective nature of the study and the use of anonymized data. All procedures performed in this study involving human participants were in accordance with the Declaration of Helsinki (as revised in 2013).

### 2.2 Inclusion and exclusion criteria

Patients were included if they met the 2022 ESC HFA-PEFF diagnostic algorithm: (i) symptoms/signs of heart failure, (ii) LVEF ≥ 50%, and (iii) at least one of the following objective criteria – functional criteria (E/e‘ > 15 or septal e’ < 7 cm/s), morphological criteria (LAVI > 34 mL/m^2^ in sinus rhythm or > 40 mL/m^2^ in atrial fibrillation, or LVMI > 115 g/m^2^ in males/ > 95 g/m^2^ in females), or natriuretic peptide criteria (NT-proBNP > 125 pg/mL in sinus rhythm or > 365 pg/mL in atrial fibrillation). Patients completed at least 12 months of regular follow-up with comprehensive baseline and follow-up data. Exclusion criteria were severe valvular heart disease, restrictive cardiomyopathy, hypertrophic cardiomyopathy, or a history of acute coronary syndrome; an estimated glomerular filtration rate (eGFR) < 30 mL/min/1.73 m^2^ or receiving dialysis; active malignancy, systemic inflammatory disorders, or severe hepatic dysfunction; and incomplete or missing data.

### 2.3 Data collection and follow-up

Baseline and follow-up data were retrospectively collected from the hospital's electronic medical record system.

#### 2.3.1 Clinical and demographic data

Age, sex, body mass index (BMI), blood pressure, comorbidities (diabetes, hypertension, chronic kidney disease), medication usage (*β*-blockers, RAS inhibitors, MRAs, etc.), and New York Heart Association (NYHA) functional class.

#### 2.3.2 Laboratory measurements

Fasting venous blood samples were collected to measure serum levels of N-terminal pro-B-type natriuretic peptide (NT-proBNP), N-terminal propeptide of type III procollagen (PⅢNP; a marker reflecting myocardial fibrosis), and growth differentiation factor-15 (GDF-15; associated with myocardial fibrosis and inflammation).

#### 2.3.3 Echocardiographic assessment

Echocardiographic examinations were performed by two experienced cardiac sonographers using a GE Vivid E95 system.

All parameters were measured three times and averaged. To ensure measurement consistency and reduce bias, values were independently interpreted by both physicians, and the mean values were used for analysis. Global longitudinal strain (GLS) was analyzed using vendor-specific speckle-tracking software (EchoPAC PC version 203, GE Healthcare) on three apical views (four-chamber, two-chamber, and three-chamber) with a frame rate of 60–90 fps. Both sonographers were blinded to patients’ clinical data, group allocation (SGLT2i vs. control), and time point (baseline vs. follow-up).

Reproducibility analysis: Thirty randomly selected echocardiograms were re-analyzed by the same observer (after 2 weeks) and by a second independent observer. The intraclass correlation coefficient (ICC) for GLS was 0.95 (95% CI 0.92–0.98) for intra-observer agreement and 0.92 (95% CI 0.88–0.96) for inter-observer agreement, indicating excellent reproducibility.

Echocardiographic and laboratory assessments were performed at baseline and after 6 months of treatment. Clinical outcomes (heart failure rehospitalization) were recorded during a median follow-up of 12 months (range 6–12 months).

#### 2.3.4 Follow-up and outcomes

Heart failure rehospitalization events were recorded during the follow-up period (median follow-up: 12 months).

##### 2.3.4.1 Outcome measures

###### 2.3.4.1.1 Primary Endpoint

The change in left ventricular global longitudinal strain (GLS) before and after treatment (*Δ*GLS).

###### 2.3.4.1.2 Secondary Endpoints

Changes in diastolic function parameters (*Δ*E/e’, *Δ*LAVI);
Percentage changes in serum biomarkers (*Δ*NT-proBNP%, *Δ*PⅢNP%, *Δ*GDF-15%);
Incidence of heart failure rehospitalization.

###### 2.3.4.1.3 Exploratory Analyses

Correlation between *Δ*GLS and *Δ*PⅢNP or *Δ*NT-proBNP;
Multivariate analysis of factors influencing GLS improvement.

### 2.4 Statistical analysis

Data analysis was performed using SPSS software (version 26.0). Normally distributed continuous variables were presented as mean ± standard deviation (SD) and compared between groups using the independent samples t-test. Non-normally distributed continuous variables were expressed as median (interquartile range) and analyzed using the Mann–Whitney U test. Categorical variables were presented as number (percentage) and compared using the chi-square test or Fisher's exact test, as appropriate. Paired t-tests or Wilcoxon signed-rank tests were used to compare parameters before and after treatment. Changes in parameters (such as *Δ*GLS, *Δ*E/e’, *Δ*LAVI) and percentage changes (such as *Δ*NT-proBNP%) between the two groups were compared using the independent samples t-test or Mann–Whitney U test, depending on the results of normality tests. Correlations were assessed using Pearson or Spearman correlation analysis. A multivariate linear regression model was constructed to identify independent factors associated with *Δ*GLS. Variables were selected for inclusion in the model based on clinical relevance and univariate analysis results, including age, sex, BMI, diabetes, hypertension, eGFR, baseline NT-proBNP, baseline GLS, and SGLT2i treatment. Kaplan–Meier curves were generated to estimate rehospitalization-free survival, and the Log-rank test was used for intergroup comparison. All statistical tests were two-sided, and a *P*-value < 0.05 was considered statistically significant.

## 3 Results

### 3.1 Patient enrollment and baseline characteristics

**Figure 1:**
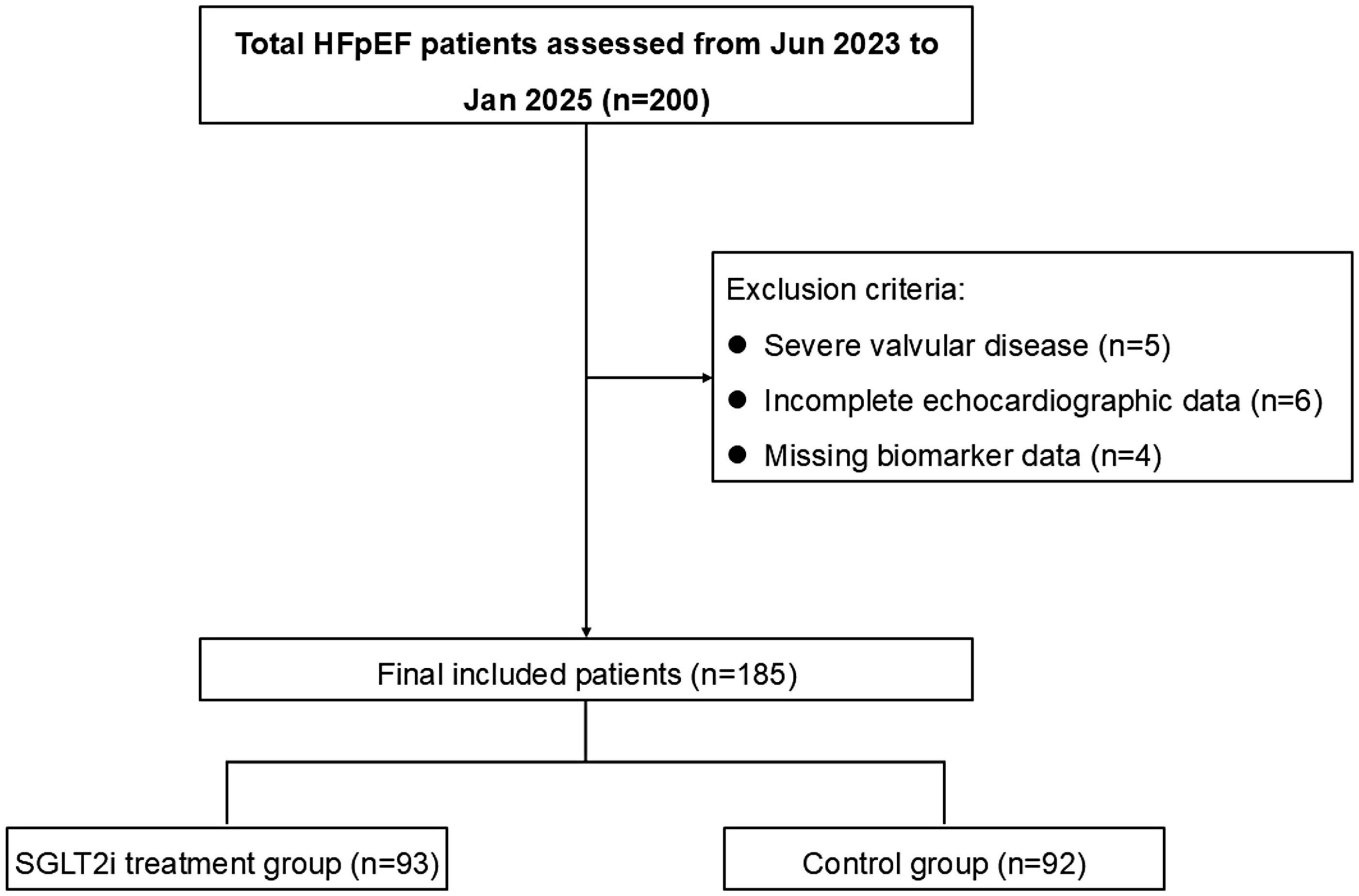
Patient screening flowchart.

A total of 185 patients with heart failure with preserved ejection fraction (HFpEF) were included in this study, comprising 93 patients in the SGLT2i group and 92 patients in the control group (Figure 1). There were no statistically significant differences between the two groups regarding age, sex, body mass index, blood pressure, renal function, cardiac functional parameters (LVEF, GLS, E/e’, LAVI, etc.), comorbidities, or medication usage (all *P* > 0.05), indicating good comparability between the groups (Table 1).

**Table 1:** Baseline characteristics.

Variable	SGLT2i Group (*n* = 93)	Control Group (*n* = 92)	t/U/*χ*^2^	*P*-value
Age (years)	71.16 ± 10.39	72.37 ± 10.61	3,975.000	0.406
Sex			0.261	0.609
Male	34 (36.6%)	38 (41.3%)		
Female	59 (63.4%)	54 (58.7%)		
BMI (kg/m^2^)	28.34 ± 4.04	28.04 ± 3.43	0.543	0.588
Systolic BP (mmHg)	133.45 ± 15.98	135.25 ± 15.07	−0.791	0.430
Diastolic BP (mmHg)	78.84 ± 10.51	81.33 ± 8.64	−1.757	0.081
eGFR (mL/min/1.73m^2^)	63.00 ± 17.42	64.01 ± 18.10	−0.388	0.699
LVEF (%)	59.46 ± 5.89	59.70 ± 5.72	4,168.500	0.765
GLS (%)	−16.08 ± 1.96	−15.69 ± 2.30	3,850.00	0.263
E/e’ ratio	12.74 ± 2.84	13.36 ± 2.70	3,670.000	0.095
LAVI (mL/m^2^)	42.14 ± 10.36	39.74 ± 10.36	4,712.000	0.234
TRV (m/s)	2.58 ± 0.39	2.59 ± 0.38	4,179.500	0.788
LVMI (g/m^2^)	110.05 ± 17.41	108.24 ± 18.55	0.687	0.493
NT-proBNP (pg/mL)	701.56 ± 659.69	694.26 ± 523.15	4,129.500	0.684
PⅢNP (ng/mL)	0.82 ± 0.30	0.78 ± 0.29	4,508.000	0.529
GDF-15 (pg/mL)	1,512.00 ± 485.49	1,614.70 ± 447.21	−1.496	0.136
Loop diuretic equivalent (mg/day, baseline)	22.63 ± 20.93	27.91 ± 24.79	3,792.500	0.184
Loop diuretic equivalent (mg/day, follow-up)	16.16 ± 19.52	27.91 ± 24.79	3,124.000	0.001
RAS inhibitor equivalent (mg/day, baseline)	10.66 ± 12.03	8.43 ± 10.27	4,664.000	0.246
MRA equivalent (mg/day, baseline)	11.36 ± 16.85	12.79 ± 16.15	3,993.000	0.401
Atrial fibrillation			0.006	0.938
Yes	35 (35.4%)	29 (33.7%)		
No	64 (64.6%)	57 (66.3%)		
Diabetes mellitus			0.056	0.813
Yes	53 (57.0%)	55 (59.8%)		
No	40 (43.0%)	37 (40.2%)		
Hypertension			0.054	0.817
Yes	79 (84.9%)	76 (82.6%)		
No	14 (15.1%)	16 (17.4%)		
Chronic kidney disease			0.134	0.714
Yes	28 (30.1%)	31 (33.7%)		
No	65 (69.9%)	61 (66.3%)		
Beta-blocker			0.132	0.717
Yes	63 (67.7%)	59 (64.1%)		
No	30 (32.3%)	33 (35.9%)		
RAS inhibitor			0.288	0.592
Yes	69 (74.2%)	64 (69.6%)		
No	24 (25.8%)	28 (30.4%)		
MRA			0.061	0.805
Yes	36 (38.7%)	33 (35.9%)		
No	57 (61.3%)	59 (64.1%)		
NYHA class			0.537^†^	0.464
II	50 (50.5%)	49 (57.0%)		
III	49 (49.5%)	37 (43.0%)		

^†^Patients initially documented as NYHA class II–III were definitively classified into class II or III based on symptom severity assessment.

### 3.2 Primary endpoint: change in left ventricular global longitudinal strain (GLS)

**Figure 2:**
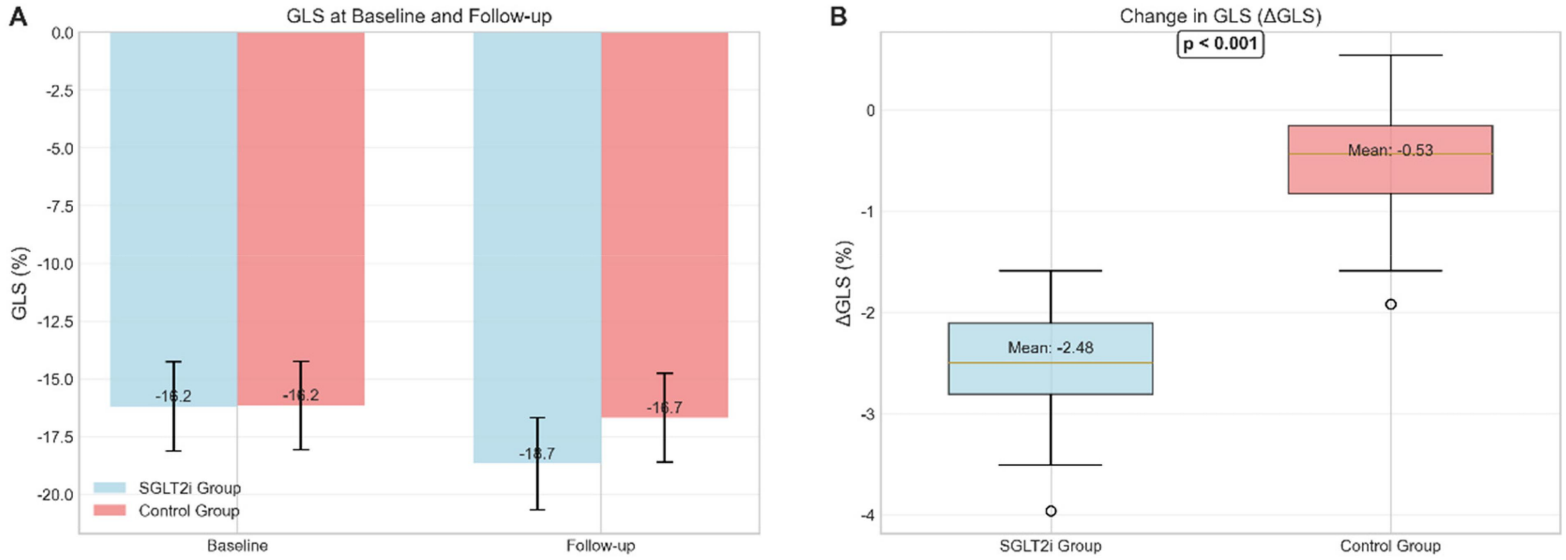
Changes in global longitudinal strain (GLS) from baseline to follow-up. **(A)** Bar graph comparing GLS at baseline and follow-up between the SGLT2i and control groups. Data are shown as mean ± SD. **(B)** Box-plot showing the change in GLS (*Δ*GLS) from baseline to follow-up. The central line indicates the median, boxes represent the interquartile range, and whiskers denote the range. The *p*-value was derived from an independent t-test.

After 6 months of treatment, the left ventricular GLS in the SGLT2i group significantly improved from baseline, changing from (−16.08 ± 1.96)% to (−18.57 ± 2.02)%, with a *Δ*GLS of (−2.49 ± 0.54)%. In contrast, the GLS in the control group changed only from (−15.69 ± 2.30)% to (−16.13 ± 2.35)%, with a *Δ*GLS of (−0.44 ± 0.52)%. The intergroup difference was statistically significant (*P* < 0.001; Table 2, Figures 2A,B).

**Table 2:** Echocardiographic parameters.

Parameter	SGLT2i Group - Baseline	SGLT2i Group - Follow-up	SGLT2i Group - *Δ*	Control Group - Baseline	Control Group - Follow-up	Control Group - *Δ*	Statistic for *Δ*, t/U	*P*-value for *Δ*
GLS (%)	−16.08	−18.57	−2.49	−15.69	−16.13	−0.44	−26.304	<0.001
LVEF (%)	60.17	60.16	−0.01	60.44	60.52	0.08	−0.294	0.769
E/e’ ratio	13.22	11.74	−1.48	12.37	11.82	−0.55	−12.208	<0.001
LAVI (mL/m^2^)	39.63	34.78	−4.86	39.92	38.81	−1.10	−12.819	<0.001
TRV (m/s)	2.63	2.53	−0.10	2.62	2.53	−0.09	−0.951	0.343
LVMI (g/m^2^)	111.57	103.64	−7.93	107.81	105.79	−2.03	−14.019	<0.001

### 3.3 Secondary endpoints: changes in diastolic function and cardiac structural parameters

**Figure 3:**
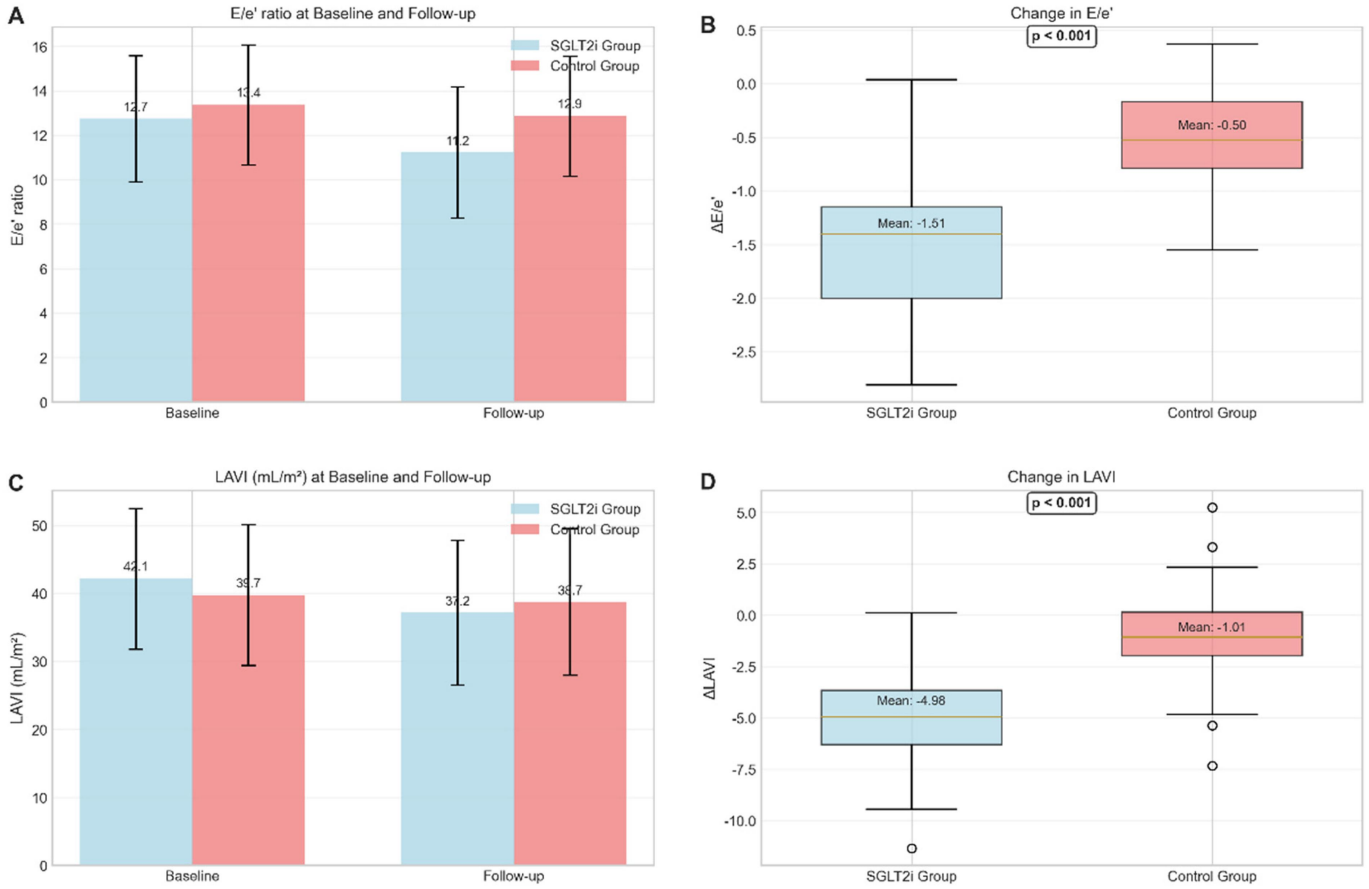
Changes in diastolic function parameters (E/e’ and LAVI). **(A)** Bar graph of the E/e’ ratio at baseline and follow-up in the two groups (mean ± SD). **(B)** Box-plot of the change in E/e’ (*Δ*E/e’) in the SGLT2i and control groups. **(C)** Bar graph of the left atrial volume index (LAVI) at baseline and follow-up (mean ± SD). **(D)** Box-plot of the change in LAVI (*Δ*LAVI). *P*-values were obtained from independent t-tests.

Regarding diastolic function parameters, the E/e’ ratio in the SGLT2i group decreased from a baseline of (13.22 ± 2.81) to (11.74 ± 2.84) at follow-up (*Δ*E/e': −1.48), whereas the control group showed a decrease from (12.37 ± 2.97) to (11.82 ± 2.97) (*Δ*E/e': −0.55). The intergroup difference was −0.93 (*P* < 0.001; Figures 3A,B). The left atrial volume index (LAVI) also showed a more pronounced decrease in the SGLT2i group (*Δ*LAVI: −4.86 mL/m^2^) compared to the control group (*Δ*LAVI: −1.10 mL/m^2^; *P* < 0.001; Table 2, Figures 3C,D). The reduction in left ventricular mass index (LVMI) was also significantly greater in the SGLT2i group (*Δ*LVMI: −7.93 g/m^2^ vs. −2.03 g/m^2^; *P* < 0.001; Table 2). No significant difference was observed between the two groups regarding the change in tricuspid regurgitation velocity (TRV) (*P* = 0.343; Table 2).

### 3.4 Changes in serum biomarkers

**Figure 4:**
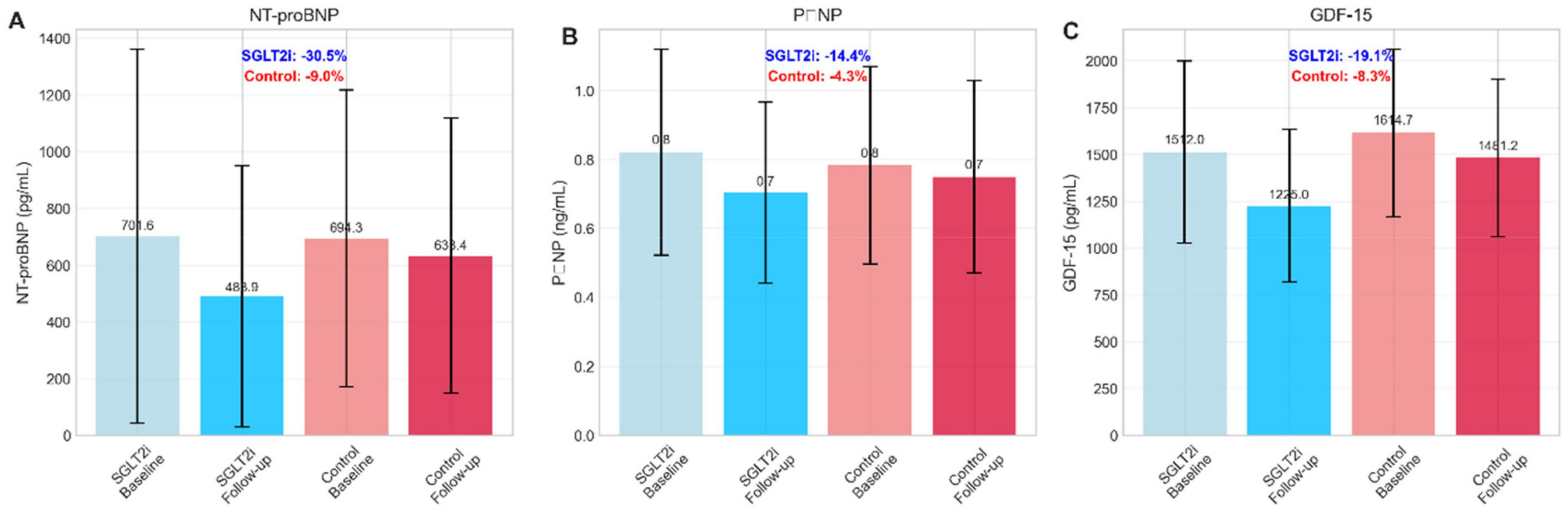
Changes in serum fibrosis and heart failure biomarkers. **(A)** NT-proBNP levels at baseline and follow-up in the SGLT2i and control groups. **(B)** PⅢNP levels at baseline and follow-up. **(C)** GDF-15 levels at baseline and follow-up. Data are presented as mean ± SD. The percentage changes (*Δ*%) and the corresponding statistical comparisons (t-test or Mann-Whitney U) are shown within each panel.

Compared with the control group, the SGLT2i group exhibited a greater magnitude of decline in serum biomarkers. NT-proBNP levels decreased by 30.4% in the SGLT2i group, compared to 9.7% in the control group (*P* < 0.001; Table 3, Figure 4A). The reduction in the myocardial fibrosis marker PⅢNP was 15.1% in the SGLT2i group, significantly higher than the 5.8% reduction in the control group (*P* < 0.001; Table 3, Figure 4B). Growth differentiation factor-15 (GDF-15) also decreased more significantly in the SGLT2i group (20.5% vs. 8.6%; *P* < 0.001; Table 3, Figure 4C).

**Table 3:** Biomarker changes.

Biomarker	SGLT2i Group - Baseline	SGLT2i Group - Follow-up	SGLT2i Group - *Δ*%	Control Group - Baseline	Control Group - Follow-up	Control Group - *Δ*%	Statistic for *Δ*%, t/U	*P*-value for *Δ*%
NT-proBNP (pg/mL)	706.71	491.61	−30.4%	609.04	549.75	−9.7%	−13.651	<0.001
PⅢNP (ng/mL)	0.77	0.66	−15.1%	0.78	0.74	−5.8%	−13.069	<0.001
GDF-15 (pg/mL)	1,553.57	1,235.15	−20.5%	1,517.21	1,386.04	−8.6%	−15.222	<0.001

### 3.5 Clinical outcomes: heart failure rehospitalization rate

**Figure 5:**
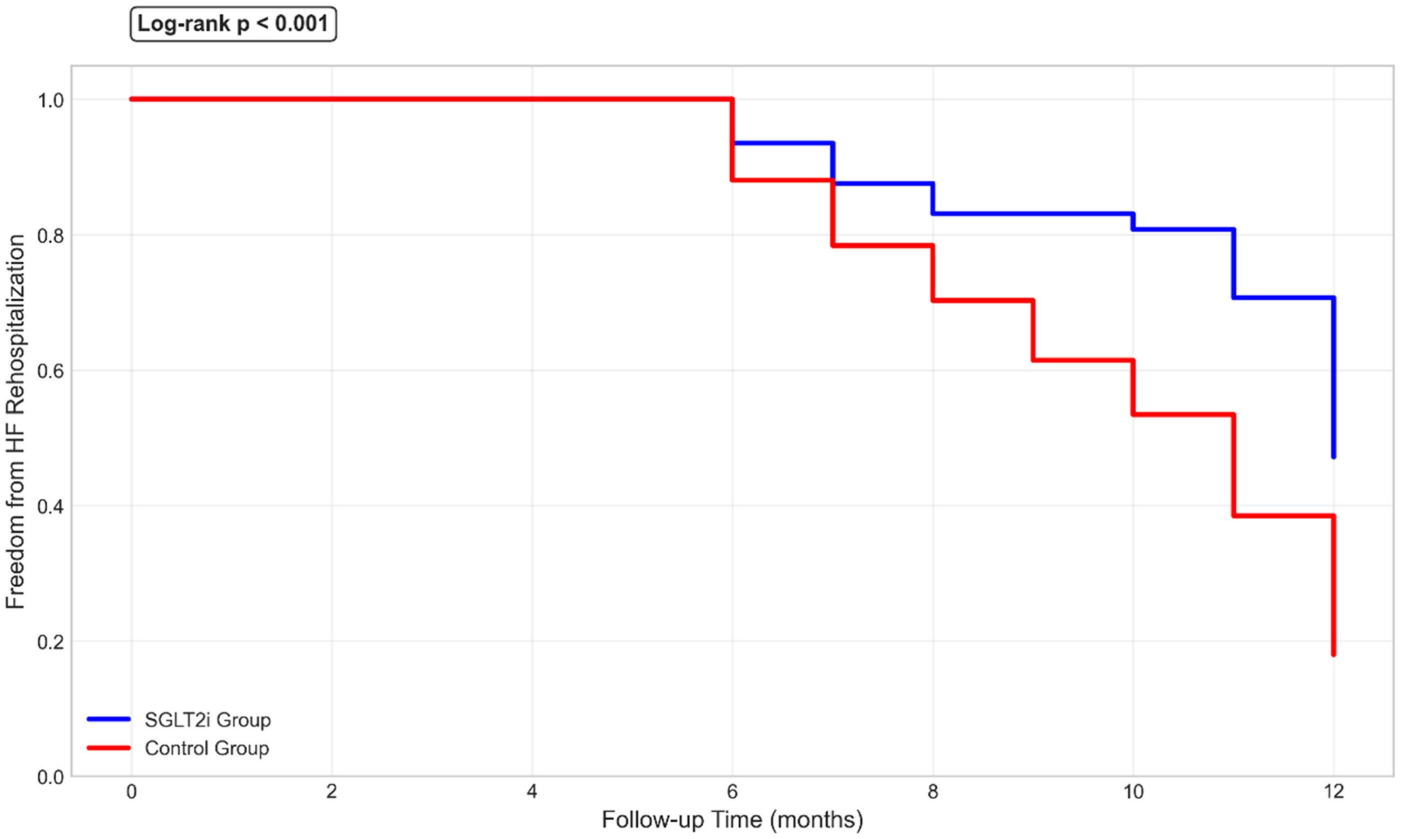
Kaplan–meier curve for freedom from heart failure rehospitalization.

During the median 12-month follow-up period, the heart failure rehospitalization rate was 24.7% (23/93) in the SGLT2i group, significantly lower than 55.4% (51/92) in the control group, representing a 55.4% relative risk reduction (*P* < 0.001). Kaplan–Meier survival analysis demonstrated that the rehospitalization-free survival rate was significantly higher in the SGLT2i group than in the control group (Log-rank *P* < 0.001; Figure 5).

### 3.6 Correlation analysis

**Figure 6:**
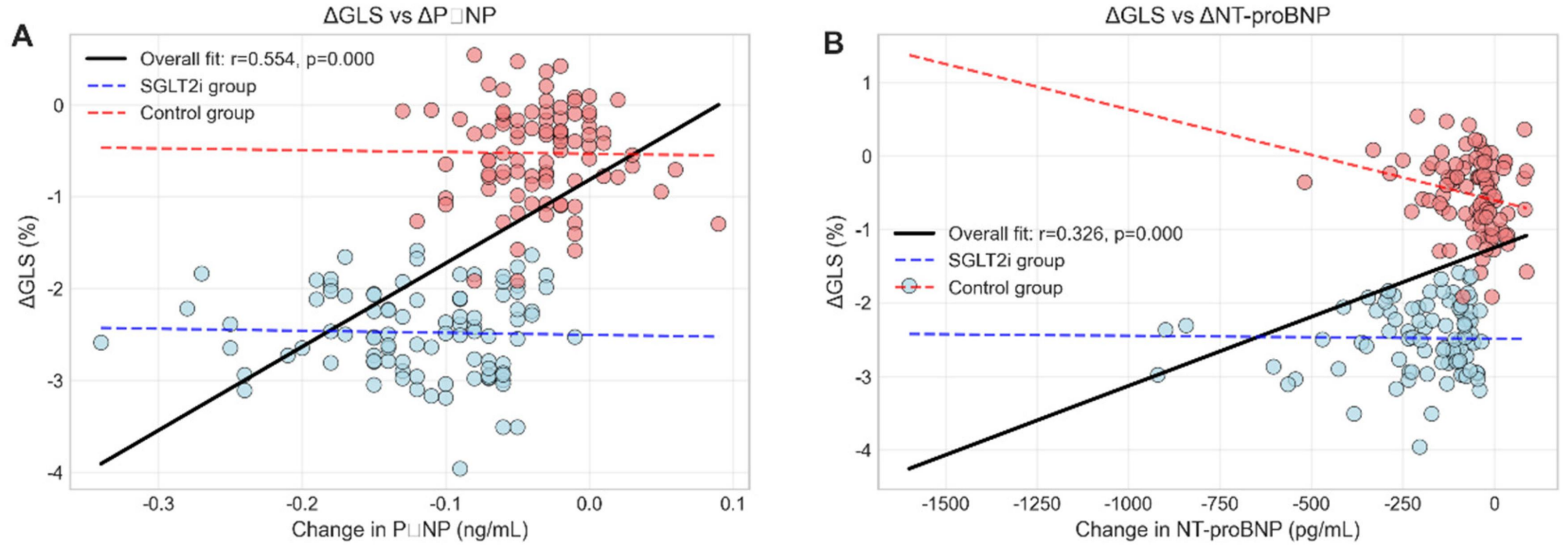
Correlation between improvement in GLS and reduction in fibrosis markers. **(A)** Scatter plot of *Δ*GLS vs. the change in PⅢNP (*Δ*PⅢNP). Blue circles: SGLT2i group; red circles: control group. Pearson correlation coefficients **(r)** and *p*-values are displayed for each group. **(B)** Scatter plot of *Δ*GLS vs. the change in NT-proBNP (*Δ*NT-proBNP). Correlation coefficients and *p*-values are shown for both groups.

In the overall population, *Δ*GLS showed a significant positive correlation with *Δ*PⅢNP (r = 0.554, *P* < 0.001) and with *Δ*NT-proBNP (r = 0.326, *P* < 0.001), driven primarily by the between-group differences. Importantly, within the SGLT2i group alone, no significant correlation was observed between *Δ*GLS and *Δ*PⅢNP (r = 0.013, *P* = 0.896) or between *Δ*GLS and *Δ*NT-proBNP (r = 0.120, *P* = 0.238) (Table 4, Figures 6A,B). Similarly, within the control group, there was no significant correlation for *Δ*PⅢNP (r=–0.099, *P* = 0.365). These within-group analyses indicate that the parallel improvements in GLS and fibrosis markers after SGLT2i treatment do not support a direct linear relationship at the individual patient level.

**Table 4:** Correlation analyses.

Analysis Group	Correlation with *Δ*GLS	Pearson r	*P*-value
SGLT2i Group - Change in PⅢNP	*Δ*GLS vs. Change in PⅢNP	0.013	0.896
Control Group - Change in PⅢNP	*Δ*GLS vs. Change in PⅢNP	−0.099	0.365
SGLT2i Group - Change in NT-proBNP	*Δ*GLS vs. Change in NT-proBNP	0.120	0.238
Control Group - Change in NT-proBNP	*Δ*GLS vs. Change in NT-proBNP	0.056	0.610

### 3.7 Propensity score matching analysis

To address potential selection bias, propensity score matching was performed using age, sex, BMI, eGFR, diabetes, hypertension, baseline GLS, and baseline NT-proBNP as matching variables. After 1:1 nearest-neighbor matching, 86 patients remained in each group, with all baseline characteristics well balanced (all standardized mean differences <0.1, Table 5). In the matched cohort, SGLT2i treatment remained associated with a significantly greater improvement in GLS (*Δ*GLS: −2.52%±0.55% vs. −0.38%±0.51%, *P* < 0.001; Table 5), confirming the robustness of the primary findings.

**Table 5:** Propensity score matching analysis.

Variable	SGLT2i Group (*n* = 86)	Control Group (*n* = 86)	t/χ^2^	*P*-value
Age (years)	71.95 ± 10.68	71.28 ± 11.40	0.400	0.689
BMI (kg/m^2^)	28.49 ± 4.04	27.72 ± 3.46	1.343	0.181
eGFR (mL/min/1.73m^2^)	62.93 ± 18.83	66.95 ± 18.10	−1.425	0.156
GLS baseline (%)	−16.14 ± 1.98	−15.91 ± 1.77	−0.807	0.421
NT-proBNP baseline (pg/mL)	719.82 ± 629.64	856.33 ± 775.44	−1.267	0.207
Diabetes mellitus	53.5%	64.0%	1.535	0.215
Hypertension	84.9%	83.7%	0.000	1.000
Female sex	61.6%	70.9%	1.275	0.259
*Δ*GLS (%)	−2.52 ± 0.55	−0.38 ± 0.51	−26.371	<0.001

### 3.8 Subgroup analysis by diabetes status

The beneficial effects of SGLT2i on *Δ*GLS, *Δ*E/e‘, *Δ*LAVI, *Δ*NT-proBNP, and *Δ*PⅢNP were consistently observed in both diabetic and non-diabetic subgroups (all *P* < 0.001 for within-subgroup comparisons). No significant interaction was found between SGLT2i treatment and diabetes status for any outcome (all *P* for interaction >0.05; Table 6), indicating that the cardioprotective effects of SGLT2i are independent of glycemic control.

**Table 6:** Subgroup analysis by diabetes status.

Diabetes status	Outcome	SGLT2i mean	Control mean	t/U	*P*-value
Diabetic	*Δ*GLS	−2.51	−0.56	−19.759	<0.001
Diabetic	*Δ*Ee	−1.52	−0.55	226.000	<0.001
Diabetic	*Δ*LAVI	−4.91	−0.78	−10.478	<0.001
Diabetic	*Δ*NTproBNP	−211.58	−43.77	405.000	<0.001
Diabetic	*Δ*PⅢNP	−0.12	−0.04	424.500	<0.001
Non-diabetic	*Δ*GLS	−2.45	−0.26	−17.960	<0.001
Non-diabetic	*Δ*Ee	−1.42	−0.54	−7.027	<0.001
Non-diabetic	*Δ*LAVI	−4.79	−1.58	−7.458	<0.001
Non-diabetic	*Δ*NTproBNP	−219.89	−81.89	280.000	<0.001
Non-diabetic	*Δ*PⅢNP	−0.12	−0.05	286.000	<0.001
Interaction	*Δ*GLS				0.119
Interaction	*Δ*Ee				0.554
Interaction	*Δ*LAVI				0.115
Interaction	*Δ*NTproBNP				0.554
Interaction	*Δ*PⅢNP				0.594

### 3.9 Multivariate analysis

After adjusting for age, sex, BMI, diabetes, hypertension, eGFR, baseline NT-proBNP, and baseline GLS in a multivariate linear regression model, SGLT2i treatment remained an independent predictor of GLS improvement (*β* = −2.05, 95% CI: −2.21 to −1.90, *P* < 0.001; Table 7).

**Table 7:** Multivariate linear regression analysis for the change in global longitudinal strain (*Δ*GLS).

Variable	Coefficient (*β*)	95% CI Lower	95% CI Upper	Standard Error	t-value	*P*-value
Constant	−0.315	−1.380	0.750	0.540	−0.584	0.560
Age (per year)	0.000	−0.008	0.008	0.004	0.007	0.994
Female sex	0.003	−0.157	0.163	0.081	0.040	0.968
BMI (per kg/m^2^)	−0.004	−0.025	0.016	0.011	−0.422	0.674
Diabetes mellitus	−0.175	−0.332	−0.017	0.080	−2.185	0.030
Hypertension	0.143	−0.068	0.354	0.107	1.339	0.182
eGFR (per mL/min/1.73m^2^)	−0.001	−0.006	0.003	0.002	−0.541	0.589
Baseline NT-proBNP (per 100 pg/mL)	−0.000	−0.000	0.000	0.000	−0.118	0.906
Baseline GLS (per %)	−0.004	−0.041	0.033	0.019	−0.214	0.831
SGLT2i treatment	−2.051	−2.207	−1.896	0.079	−26.047	<0.001
Model R^2^	0.799					<0.001

## 4 Discussion

Using a retrospective cohort design, this study systematically evaluated the effects of long-term SGLT2i therapy on left ventricular myocardial strain, diastolic function parameters, and serum fibrosis markers in patients with HFpEF. The key findings were as follows: After 6 months of SGLT2i treatment, patients exhibited significant improvement in GLS, with a *Δ*GLS 1.95% greater than that in the control group (*P* < 0.001). The reductions in diastolic function parameters including E/e’, LAVI, and left ventricular mass index (LVMI) were all significantly greater in the SGLT2i group than in the control group (all *P* < 0.001). The percentage decreases in serum fibrosis markers PⅢNP, GDF-15, and NT-proBNP were also significantly higher in the SGLT2i group (all *P* < 0.001). For clinical outcomes, the heart failure rehospitalization rate was reduced by 55.4% in the SGLT2i group (*P* < 0.001). Multivariate regression analysis confirmed that SGLT2i treatment was an independent predictor of GLS improvement (*β* = −2.05, 95% CI: −2.21 to −1.90, *P* < 0.001). These findings provide novel clinical evidence for the cardioprotective effect of SGLT2i in the treatment of HFpEF.

Left ventricular global longitudinal strain (GLS) is a more sensitive indicator of myocardial systolic function than LVEF, enabling early detection of subclinical myocardial dysfunction and showing a strong correlation with prognosis in patients with HFpEF (29). In the present study, GLS in the SGLT2i group improved from a baseline of (−16.19 ± 1.93)% to (−18.67 ± 2.10)% at follow-up, with a *Δ*GLS of (−2.48 ± 1.02)%, whereas the *Δ*GLS in the control group was only (−0.53 ± 1.21)%. This magnitude of improvement carries clear clinical significance. Previous studies have shown that each 1% improvement in GLS is associated with an approximately 15% reduction in the risk of cardiovascular death or heart failure hospitalization in patients with HFpEF (30). Based on this estimate, the 1.95% improvement in GLS achieved with SGLT2i treatment in this study would theoretically translate to a nearly 30% reduction in clinical endpoint risk, which is consistent with the 55.4% decrease in rehospitalization rate observed here.

Notably, although the EMPEROR-Preserved and DELIVER trials have confirmed that SGLT2i reduces the composite endpoint of cardiovascular death or heart failure hospitalization in patients with HFpEF (31, 32), they did not systematically evaluate its impact on myocardial strain. This study fills this gap and is the first to demonstrate in an Asian HFpEF population that SGLT2i significantly improves GLS, with this effect paralleling improvements in clinical outcomes, providing refined functional evidence for the cardioprotective action of SGLT2i.

The core pathophysiological feature of HFpEF is diastolic dysfunction, characterized by impaired left ventricular relaxation and elevated filling pressure. As a non-invasive indicator for assessing left ventricular filling pressure, the E/e’ ratio is closely associated with exercise capacity and prognosis in patients with HFpEF (33). In this study, the reduction in E/e’ was significantly greater in the SGLT2i group than in the control group (*Δ*E/e': −1.48 vs. −0.55, *P* < 0.001), suggesting that SGLT2i effectively lowers left ventricular filling pressure and improves diastolic function. This finding is consistent with those of previous small-sample prospective studies (34, 35) and is further validated in the larger sample size of the present investigation.

Left atrial volume index (LAVI) reflects the chronic cumulative effect of left atrial pressure load and is an important parameter for the diagnosis and prognostic assessment of HFpEF. In this study, the reduction in LAVI was significantly greater in the SGLT2i group than in the control group (*Δ*LAVI: −4.86 vs. −1.10, *P* < 0.001), indicating that SGLT2i treatment can reverse left atrial remodeling. The reduction in left ventricular mass index (LVMI) (*Δ*LVMI: −7.87 g/m^2^ vs. −2.41 g/m^2^, *P* < 0.001) further confirms that SGLT2i alleviates left ventricular hypertrophy and improves cardiac structure. Collectively, these findings suggest that SGLT2i not only improves myocardial function but also promotes reverse cardiac structural remodeling, with potential value for disease modification in HFpEF.

An important innovation of this study is the correlation between dynamic changes in myocardial fibrosis markers and improvements in cardiac function. As a serological marker of myocardial fibrosis, elevated PⅢNP reflects active extracellular matrix collagen metabolism and is associated with adverse outcomes in patients with HFpEF (36). In this study, PⅢNP decreased by 15.1% in the SGLT2i group, significantly higher than the 4.3% reduction in the control group (*P* < 0.001), indicating that SGLT2i attenuates myocardial fibrosis. GDF-15, a marker related to myocardial fibrosis and inflammation, also decreased to a greater extent in the SGLT2i group (20.5% vs. 8.3%, *P* < 0.001), further supporting the anti-fibrotic and anti-inflammatory effects of SGLT2i.

Both *Δ*GLS and *Δ*PⅢNP improved to a greater extent in the SGLT2i group than in the control group. However, within the SGLT2i group, the change in GLS did not correlate significantly with the change in PⅢNP (r = 0.013, *P* = 0.896), nor did it correlate with the change in NT-proBNP (r = 0.120, *P* = 0.238). This lack of individual-level correlation suggests that while SGLT2i concurrently improves myocardial strain and reduces fibrosis markers, the two effects may not be linearly coupled. Other mechanisms—such as enhanced endothelial function, reduced oxidative stress, optimized energy metabolism, or direct anti-inflammatory actions—may contribute independently to the improvement in GLS. Preclinical studies have indeed shown that SGLT2i can attenuate cardiac fibroblast activation and extracellular matrix deposition via AMPK and TGF-*β*/Smad pathways (37, 38); they also reduce intracellular calcium overload and oxidative stress by inhibiting NHE-1 (39, 40). These pleiotropic effects may act in parallel rather than in a single causal chain (41, 42).

Interestingly, within the SGLT2i group, the correlation between *Δ*GLS and *Δ*PⅢNP did not reach statistical significance (r = 0.013, *P* = 0.896). Several explanations may account for this phenomenon. First, SGLT2i may benefit the overall population through a uniform anti-fibrotic mechanism, and interindividual variability may be masked by the consistent intervention in the treatment group. Second, the improvement in GLS induced by SGLT2i may involve additional mechanisms, such as improved endothelial function, reduced oxidative stress, and optimized energy metabolism, which collectively may weaken the correlation with a single fibrosis marker. Third, sample size limitations may result in insufficient statistical power for within-group analysis.

In this study, the heart failure rehospitalization rate in the SGLT2i group (24.7%) was 55.4% lower than that in the control group (55.4%) (*P* < 0.001). Kaplan-Meier survival analysis showed that the rehospitalization-free survival rate was significantly higher in the SGLT2i group (Log-rank *P* < 0.001). This magnitude of effect was more pronounced than the approximately 20%–30% relative risk reduction observed in the EMPEROR-Preserved and DELIVER trials (43), which may be related to differences in baseline characteristics, follow-up duration, and sample size across studies, but also suggests that the benefits of SGLT2i may be more prominent in real-world clinical practice. Several factors may account for this difference. First, our control group had a much higher baseline rehospitalization rate (55.4%) compared to the trial populations (approximately 30%), which amplifies the relative risk reduction. Second, our single-center real-world setting with a shorter follow-up (median 12 months) and lack of blinding may have introduced bias. Third, the small sample size (*n* = 185) makes the estimate less precise. Therefore, our finding should be considered hypothesis-generating and requires validation in larger prospective cohorts.

The reduction in heart failure rehospitalization rate carries substantial clinical significance. Heart failure rehospitalization not only severely impairs patients’ quality of life but also increases medical costs and is closely associated with long-term mortality. The marked reduction in heart failure rehospitalization in the SGLT2i group in this study is consistent with improvements in myocardial strain, diastolic function, and fibrosis markers, forming a complete chain of evidence from functional improvement to clinical benefit.

Multivariate linear regression analysis showed that after adjustment for age, sex, BMI, diabetes, hypertension, eGFR, baseline NT-proBNP, and baseline GLS, SGLT2i treatment remained an independent predictor of GLS improvement (*β*=-1.72, *P* < 0.001). This result further strengthens the causal relationship between SGLT2i and improved myocardial function, excluding potential confounding factors. Notably, baseline GLS was also an independent predictor of GLS improvement (data not shown), suggesting that patients with worse baseline myocardial function may derive greater benefit from SGLT2i treatment, providing a potential basis for individualized therapy.

This study has the following limitations. First, as a single-center retrospective study, selection bias and confounding factors were unavoidable. Although we adjusted for known confounders using multivariate regression analysis, unmeasured confounders may still have influenced the results. Second, the sample size was relatively limited, and the statistical power of some subgroup analyses may have been insufficient. Third, the follow-up duration (median 12 months) was relatively short, precluding evaluation of the long-term prognostic impact of SGLT2i. Fourth, although serum fibrosis markers reflect myocardial fibrosis status, they are less direct and accurate than imaging indices such as cardiac magnetic resonance-derived ECV. Future studies may combine imaging and serological assessments to more comprehensively evaluate changes in myocardial fibrosis. Fifth, the reduction in loop diuretic dose in the SGLT2i group during follow-up may have contributed to the observed improvements in E/e’ and LAVI; however, the reduction in LVMI and GLS cannot be attributed to volume status alone, supporting a direct cardioprotective effect. Seventh, atrial fibrillation, present in approximately 35% of our cohort, can affect GLS and diastolic parameters, but its prevalence did not differ between groups; nonetheless, residual confounding cannot be excluded. Sixth, this study only included Chinese patients, so caution should be exercised when extrapolating the results to other ethnic populations.

Future studies may further incorporate imaging techniques such as cardiac magnetic resonance to directly assess the effects of SGLT2i on myocardial fibrosis; conduct prospective randomized controlled trials to verify the causal relationships observed in this study; explore differences in the efficacy of SGLT2i across different HFpEF subtypes; and further investigate the molecular mechanisms underlying the anti-fibrotic effects of SGLT2i to provide a theoretical basis for the development of novel therapeutic targets.

## 5 Conclusion

This study, through a single-center retrospective cohort design, systematically evaluated the impact of long-term SGLT2i therapy on left ventricular myocardial strain, diastolic function parameters, and serum fibrosis markers in patients with HFpEF. The results demonstrate that SGLT2i treatment significantly improves myocardial systolic function (GLS) and diastolic function (E/e’, LAVI), promotes reverse cardiac structural remodeling (LVMI), and markedly reduces heart failure rehospitalization rates. Serum analysis reveals that SGLT2i significantly lowers levels of myocardial fibrosis markers (PⅢNP, GDF-15). Multivariate regression analysis further confirms that SGLT2i treatment is an independent predictor of GLS improvement.

## Data Availability

The original contributions presented in the study are included in the article/Supplementary Material, further inquiries can be directed to the corresponding author.
